# Notes Taken from Lectures of M. Trousseau, on Croup and Diphtherite

**Published:** 1853-05

**Authors:** 


### ECLECTIC DEPARTMENT,

### AND SPIRIT OF THE MEDICAL PERIODICAL PRESS

---

*Notes taken from Lectures of M. Trousseau, on Croup and Diphtherite, at L'Hopital Des Enfants Malades.* By WALTER ATLEE, M. D., of Philadelphia.

PARIS, 1852.

In the Hospital, gentlemen, it is but rarely that you can follow a case of croup from one end to the other; generally you see but the last scene. The croup is the most usually curable when the physician is called in time. There is an inflammation of the mucous membrane of the larynx, sudden, acute, considerable, without false membrane, as the inflammation of the nasal mucous membrane. Let us now pass from this croup that gets well to the croup that kills. The ulcus Egyptiacum of the ancients is the same affection. The Spaniards of the sixteenth century have described it marvelously. I am going to describe an epidemic: After the fall of Napoleon, the Bourbons made what might be called provincial armies; each province furnished its legion. The legion of La Vendee was at Tours, in 1818. M. Bretonneau is there. It was observed that all the men of this legion had an affection of the gums; they were swollen, and with a white border; in some cases the cheeks and even the pharynx were covered with false membranes, white and fetid. A certain number died, soon after, in the neighborhood of the barracks, the children died of croup. M. Bretonneau, struck by this, examined what he was calling gangrenous angina, and found it to be the same affection.

I will describe to you a case of croup: An adult complains of sore throat; this year I have seen four or five cases in adults. There is a slight febrile movement and a swelling of the glands at the angle of the jaw. If you look at the tonsils you will see a piece of false membrane; an hour after it is larger, for it spreads as oil upon cloth. If you remove it, you see that it has a thickness of three or five millimetres; you see false membrane on the other tonsil, on the palate, &c. By forcibly pushing down the tongue, you can almost always see the limit of the disease; but the false membranes commence to thicken; they form a kind of stratifications, fetid, gray, brown, yellowish, gangrenous, but there is no gangrene, and it will yield in a few days to a proper medicine. Cough commences, the respiration begins to be difficult and hissing. Sometimes they get rid by the cough of false membranes, which gives an immense relief, but in twelve hours all comes back again. Let us take, now, a child of five years; he cannot tell us that he has sore throat, &c., at the commencement. He dies two or three days after the commencement of the laryngeal affection, but eight days after the affection of the pharynx. Bring up your children in the fear of the croup, and teach them to tell you when they have sore throat, and to show their throats when asked. The parents only have alarm when the cough commences. In the child the disease marches more rapidly than with the adult; with them it is very rare that the false membrane has a gangrenous appearance. This is because they do not acquire sufficient thickness. They remain white. This is the reason why in the child this same disease has one name, and in the adult, another.

In the former, the formation of false membrane may begin in the pharynx: it is, however, rarely limited to this spot, but extends with the greatest facility into the air passages, when it is called croup. In the latter, it is commonly limited to the pharynx and is then termed diphtherite.

Now I will describe the true croup: After three or four days of malaise, the child has a slight cold: moreover, he coughs a little, but the cough has no extraordinary character. After twenty-four hours the cough assumes another character; it is *hoarse* and *frequent*. The hoarseness increases with the frequency; it is very sonorous, very noisy. The voice is almost extinguished; after some days the cough becomes less hoarse, less sonorous, and much less frequent. The voice is extinguished, and the respiration assumes a peculiar character. In an individual in good health the respiration is performed without noise, but when there is a narrowing of the larynx, the air is forced to traverse it with a hissing sound. At this period the respiration is constantly difficult, and what are called paroxysms of suffocation occur; all of a sudden the difficulty of breathing becoming greater. These paroxysms at first take place every two hours, afterward every hour, then every half hour, &c. The child is so agitated in these paroxysms that sometimes there is a kind of madness. When they are over, he falls into an extreme prostration. He dies, generally, half an hour after a paroxysm that has left him totally prostrated, cyanosed. It is very rare that he dies in a paroxysm.

This disease has then a very slow march; but, nevertheless, in some very rare cases, from the commencement of the alteration of the voice to the death, but twenty-four hours elapse. Ordinarily there is an interval of five, six, or seven days.

Meanwhile this cough, that had been hoarse and frequent, from the moment that the oppression begins to be felt, loses its hoarseness and its frequency. Ordinarily it is extinguished and hissing, very rarely hoarse, never frequent.

They speak of premonitory symptoms of this disease, but the fever, the swelling of the glands at the angle of the jaws, are not premonitory symptoms of the disease, but of the affection of the larynx.

Before these false membranes are formed, something is necessary,—a phlegmasia. When this phlegmasia commences it has no particular anatomical character. Do not then be surprised that you have the same characters in the cough and in the voice, as in a common catarrh. When the ligaments of the glottis are invaded, and after the phlegmasia has lasted for twenty-four hours, they are covered with false membranes. Then, in place of the ligaments being smooth and tense, they are covered by these false membranes. The voice and cough are necessarily extinguished, and the cough becomes rare. Why? Because, the ligaments being inflamed, the cold air irritated them; but when there is an interposition of false membrane, there is no irritation, and he coughs no more.

It is a disease slow in its evolution, but in some very rare cases it lasts but two or three days. The slower the disease in its evolution, the more certain is the operation of tracheotomy.

There are some cases, *exceedingly* uncommon, where the disease commences in the larynx, but for some physicians, they are not uncommon. Let me explain to you why. In the greater number of cases the physician is not called at the beginning of the affection of the pharynx, but one or two days after the affection of the larynx, and then he does not occupy himself with that. And, moreover, there are many physicians who cannot examine the throat of a child. A physician should know how—he must learn. The first thing to do is to hold firmly the head; when that is done the child begins to cry; pass quickly the spoon, and always a very large one, into the mouth, and push it into the pharynx; the child has a desire to vomit and then opens its mouth widely. You must push the spoon beyond the isthmus of the throat. When he closes firmly his teeth, hold his nose, pinch his lips, and when you loosen the lips and he opens his mouth, in order to breathe, pass quickly the spoon and force it back into the pharynx. Sometimes a child who had false membranes of the pharynx, has them no longer; but in these cases a swelling of the glands at the angle of the jaws remains.

Some events, I must tell you of, that occur and are of a nature to deceive you. After a paroxysm, a tube of false membrane is thrown off, and after that the child breathes as he does naturally. You cry victory, and in twenty-four hours he is dead. Do not be in a hurry to give a favorable prognosis, for of a hundred cases, where false membranes are thus thrown off, ninety-nine die. After he has thrown them off three or four times, give a more favorable prognosis.

Let us now commence the history of diphtheritis. It is in a village, in a hamlet, that you can study an epidemic—in this Babylon you cannot. I have studied several epidemics, and have recognized the diphtheritis to be a specific disease, having peculiar characters in all the tissues.

When the diphtheritic virus is placed in contact with the skin, it causes a false membrane. Ammonia produces the same effect, and the measles can do it. You insert under the skin some syphilitic matter, and after some days you destroy it; nothing is produced—which proves that the syphilis is at first a local disease. Let us regard some affections more acute, that are at first local. If you insert under the skin some vaccine matter, and at the end of four days you destroy the pustule with some caustic, in eight days the child will take the vaccine. The diphtheritis holds the middle ground in regard to duration. The syphilis and the vaccine are at the two ends of the ladder. It is a long time local. When I was in S——, in 1828, to study the epidemic, I saw a child who had false membranes on an abraded surface. The skin had been rubbed off, while another child who had false membranes behind the ears, was drawing him on the barrow. The one had had the false membranes a month, the other a longer time. They called the disease then the *mal blanc*, and also chancre. I saw there one woman who had taken the disease by the genital organs, one girl who had taken it by a blister, another by a scratch on the breast.

It is a disease that is not a disease of the throat, and this is why the name croup is a bad name. You say that there is a scarlatinous anasarca, a syphilitic caries. You do not say that there is an exostosis, but you say that it is the syphilis with a manifestation in the bones. You must resume the names of the disease by synthesis. The term dyphtheritis, signifies a disease with production of false membranes; but false membranes having a specific character, that can occupy all the parts of the body.

The diphtheritis remains fixed, if it occur upon the skin, as in a wound or in the vulva. It causes gangrene of the skin, but more especially is this the case when the vulva is attacked. It is rare to see gangrene when the gums are attacked, except sometimes in the hospitals.

There are several maladies that are accompanied with sore throat—for instance, the scarlatina. The sore throat, then, has great resemblance to the croup, for the tonsils are covered with a white coating, that you can well mistake for some false membranes; but that does not deceive you if you take into consideration the symptoms of the disease. In the scarlatina there is always fever, the tongue is swollen, and its papillæ become very manifest, giving it the appearance of a strawberry. In the scarlatina, the cervical glands are seized; the sore throat remains always limited to the tonsils, and does not attack the larynx and the trachea, as the croup does. In the diphtheritis the disease invariably advances, a white spot is seen on the tonsils, which increases as a drop of oil on paper.

The analysis of the false membranes of croup and those of other anginas, teaches us nothing; there is no difference. How, then, can we recognize the disease? You must see and study the premonitory symptoms and progress of the affection.

The acute laryngitis of pseudo croup is, without doubt, only a simple acute laryngitis, with a greater degree of intensity. The latter attacks young children very suddenly—very rarely adults. It is a disease sudden and rapid in its progress, without premonitory symptoms. The child goes to bed well, and perhaps at midnight wakens up with suffocation and cough. The voice is vailed; there is fever. With the day all the symptoms disappear to return the night following; and, moreover, the cough in the daytime diminishes, becomes hoarse, and it is terminated by a simple catarrh. Then there have been no false membranes. This is a malady of the kind which succeeds very well with the homœopathics, for it invariably has a happy termination.

In the true croup, the child has a malaise of three or four days; he does not eat, or very little, for it makes him suffer. There is an alteration of the voice; there is croupal cough, fetid breath, and great difficulty of deglutition. The voice remains vailed. I have seen several cases where the patients have died by disease of the pharynx, the diphtheritis having taken that road, and having limited itself exclusively to that part, without affecting, in the smallest degree, the larynx.

*Treatment.*—Every time that you see appear on the mouth or throat, whitish concretions, the first indication is the local treatment. Epidemic diphtheritis is very mortal. I have seen fifteen die in a family of sixteen. The treatment by emetics, succeeds sometimes quite well, as a mechanical means, in order to displace the false membranes. When you are called to a patient with croup, examine the mouth, and you will almost always find that the disease is limited to the pharynx. The first thing to do, is to cauterize, thoroughly, the throat with nitrate of silver. Repeat the cauterization several times a day. You must not be satisfied, until you see the tonsils red. During the cauterizations with the nitrate of silver, you must also make insufflations of alum into the throat. This insufflation of alum should be repeated several times every day. You must blow hard, without pity, for the alum can do no harm, even in great quantity. The hydrochloric acid and nitrate of silver are the best caustics; never use the sulphuric acid. If you prefer the solution of nitrate of silver, put one part of it to five of water. When the disease has invaded the larynx, the treatment becomes much more difficult. In order to cauterize the larynx well, you must have a piece of whalebone, that you curve as you wish, by placing it in hot water. To its extremity, you fasten a bit of sponge, in order to dip it into the caustic solution, and you introduce it into the larynx itself. Make the child swallow, every hour, a teaspoonful of honey, with a little calomel and alum.

In 1822, M. Bretonneau tried to perform tracheotomy; all the patients died: but they would have died any how. In the month of August, 1825, he operated upon a young girl, and was successful. In this case, he made use of a larger canula. In 1830, I had a successful case. In 1834, I operated thirty-four times, and some of the cases got well. In fine, now-a-days, the operation has passed into medical usage.

*Indications for this operation.*—By what signs do you recognize its necessity? When the affection has commenced in the pharynx, and has extended slowly, in such cases the recovery is so rare, without tracheotomy, that you must operate. You must not wait for the last moment of asphyxia. When the cough is rare, generally extinguished; when the respiration is hissing; when there are orthopnœa and the paroxysms of suffocation, then you must operate.

In what circumstances will the operation succeed the best? I believe that there is a better chance of success when the operation is performed too soon. They ask you: "But if you do perform the operation, will my child get well?" I answer: "I do n't know, at all." "But what chance?" "One chance in five." Of one hundred cases there are twenty-five successful. I have not hesitated to perform the operation upon children dead—that is to say, no longer breathing, and they got well. This extreme degree of asphyxia is an inconvenience, but it is not a formal contra-indication.

When the disease has progressed slowly, hope much. When suddenly, do not hope. When there are false membranes on blistered surfaces—the nose, &c.—it rarely if ever succeeds. As to the age, it is from three to five years that you count the greatest number of cases. With adults, you are almost always unsuccessful. Under two years of age, the operation is quite useless; they die, almost always, of convulsions.

*For the operation.*—You must have a matress, on a hard and flat table. No pillow, but a block of wood, under the neck. The instruments you require are, a common bistoury, a blunt-pointed one, some hooks, a dilator, and some canulas—the canula to be double, and its diameter as great as possible, consistently with the age of the patient; a proper number of aids; and you must have a *tallow* candle—never use one of wax—to perform an operation. The block must be placed under the first dorsal vertebra, and, perhaps, under the last cervical. The child has always the time to be operated upon slowly. trace a line with a burnt cork. Then open the skin, *perfectly at your ease.* You cut, carefully, between the muscles, until, at last, you perceive the thyrhoid gland, lying across the trachea. You cut the gland, and a small jet of arterial blood spouts; but do not trouble yourself with that. At last, you see the rings of the trachea at the bottom of the wound. Now, suppose a case in which the veins trouble you; if you cannot avoid them, cut them. The jet is violent, but soon ceases. Cut a millimetre each stroke, and use the sponge after each stroke. Ordinarily, you come upon the first ring, or the cricoid cartilage. You lay bare three rings; with the bistoury you make a small hole, the size of a pin's head; then you introduce the blunt-pointed bistoury, and finish. You make use of the dilator, and at the end of a minute you introduce the canula.

*Dangers of the Operation.*—What are the dangers that frighten the young physician, and that ought to do so? It is rather the operation of the physician, than of the surgeon; one of those little operations that a physician ought to perform. I repeat it again, that you have enough time to perform it. You must go to work, then, slowly. Of all the accidents, the gravest is the cutting of the carotid. It happens, in some very rare cases, that the carotid takes an anomalous course; but if you perform the operation as you should perform it, there is no danger even then. Twice in my life I have encountered anomalies, and I have drawn aside the vessel with a hook. The venous hæmorrhages, are they dangerous? They exhaust the child, and again they make the physician lose his presence of mind. When you have cut some veins, do not tie them—it is useless; but let me advise you to wait. Put your finger on the bottom part of the wound, sponge, and *wait.* Hold the child up; quiet him a little; *you have time enough.* You may attack the trachea too violently. It is not as large as the finger, and flexible. Be gentle, cut by very slight strokes, and then use the blunt-pointed bistoury; with it you can cut nothing else. If the child has an attack of syncope, wait. When the trachea is opened, the blood enters. This is by no means a great inconvenience; open the trachea with the dilator; the child breathes well, and the flow of blood ceases. After the operation, you often see an attack of syncope; but it is nothing. The operation is over, and the canula is introduced. Immediately after, the canula becomes a source of irritation; but after some time that passes off. The febrile excitement, &c., diminish. This state lasts seven to twelve hours, and there are false membranes in the bronchial tubes. In some cases, in twenty-five or forty hours, there is a change. In these two circumstances there is not the same cause of death. If, at the end of a quarter of an hour, there is a respiration analogous to the sound of a saw, sawing a stone—the serratic respiration—death is certain. If the pulse, at the end of twenty-four hours, becomes extremely frequent, the children die. When the child is seized with convulsions, he dies. I speak here of the signs that I have observed myself. After twenty-four hours, the wound generally swells a great deal. It is a favorable sign; if it does not swell, the children die. There is a complication, an enormous swelling of the neck. You must ward it off, by cauterizing the edges of the wound, at the end of twelve hours. You must repeat the cauterization three or four times. If this enormous swelling takes place, the children invariably die.

By a provision of nature, the air enters the nose, and is warmed, and becomes moist before entering into the lungs. On the contrary, in these cases, the air enters cold and dry. A cravat should be placed around the neck of the child, extending over the chin and the upper part of the chest. Thus, the air entering the lungs will be warm and moist. These things are so important, that I do not hesitate to say that the great number of successful cases in three years ought to be attributed to two things—the cauterization and the cravat. I was obliged to make use of the cravat, because I had to inject water to moisten the hardened mucus, and that tormented the child, and irritated the bronchial tubes.

*Presentation of false membranes.*—At the moment of finishing the operation, a gush of mucus issues, and sometimes a false membrane, occasionally bifurcated; but in a certain number of cases, on introducing your dilator, you see a white membrane, and you hear a noise when he breathes—the noise of a valve. In these cases, you should not introduce the canula. Try to excite the cough, by the introduction of some drops of water, and when the false membrane is expelled, you introduce the canula, and wait for aaother. When you have waited for three or four days, without any other accident occurring, you can believe the child cured. After that time, the danger is not of extension into the bronchial tubes, but in the lungs; for when there is an extension into the bronchial tubes, they die in twelve or fifteen hours. The danger is of lobular pneumonia. These accidents that I have just described, can be called accidents inherent in the operation.

*Other accidents.*—Sometimes, at the end of sixteen days, the child can no longer swallow; everything enters into the trachea and comes out by the canula, or enters the lungs. For this, there is but one remedy—to make them swallow solid substances, as some long vermicelli. If you have the slightest sign of diphtheritic cachexia, give your patients coffee with quinine.

When must you think of removing the canula? At the end of four or five days, you remove the canula softly, in order to see. Here, in the hospital, after its removal, they leave the wound open, covered with a little *linge fenetre*, the child being left to breathe by both openings, until the wound is closed. I think I shall adopt this plan.—*Charleston Med. Jour. and Rev.*

